# Assessment of ferroptosis as a promising candidate for metastatic uveal melanoma treatment and prognostication

**DOI:** 10.3389/fphar.2024.1466896

**Published:** 2024-10-01

**Authors:** Ellie Swords, Breandán N. Kennedy, Valentina Tonelotto

**Affiliations:** ^1^ UCD Conway Institute, University College Dublin, Dublin, Ireland; ^2^ UCD School of Biomolecular and Biomedical Science, University College Dublin, Dublin, Ireland

**Keywords:** uveal melanoma, metastasis, ferroptosis, treatment, prognosis

## Abstract

Uveal melanoma (UM) is the most common primary intraocular tumour in adults. Local resection, radiation therapy, and enucleation are the current first-line, primary UM treatments. However, regardless of the treatment received, around 50% of UM patients will develop metastatic disease within five to 7 years. In the largest published series of unselected patients with metastatic UM (mUM), the median survival time after diagnosis of metastasis was 3.6 months, with less than 1% of patients surviving beyond 5 years. Approved drugs for treatment of mUM include systemic treatment with tebentafusp-tebn or isolated hepatic perfusion (IHP) with melphalan. However, these drugs are only available to a subset of patients and improve survival by only a few months, highlighting the urgent need for new mUM treatments. Accurately predicting which patients are at high risk for metastases is also crucial. Researchers are developing gene expression signatures in primary UM to create reliable prognostic models aimed at improving patient follow-up and treatment strategies. In this review we discuss the evidence supporting ferroptosis, a non-apoptotic form of cell death, as a potential novel treatment target and prognosticator for UM.

## 1 Introduction

**FIGURE 1:**
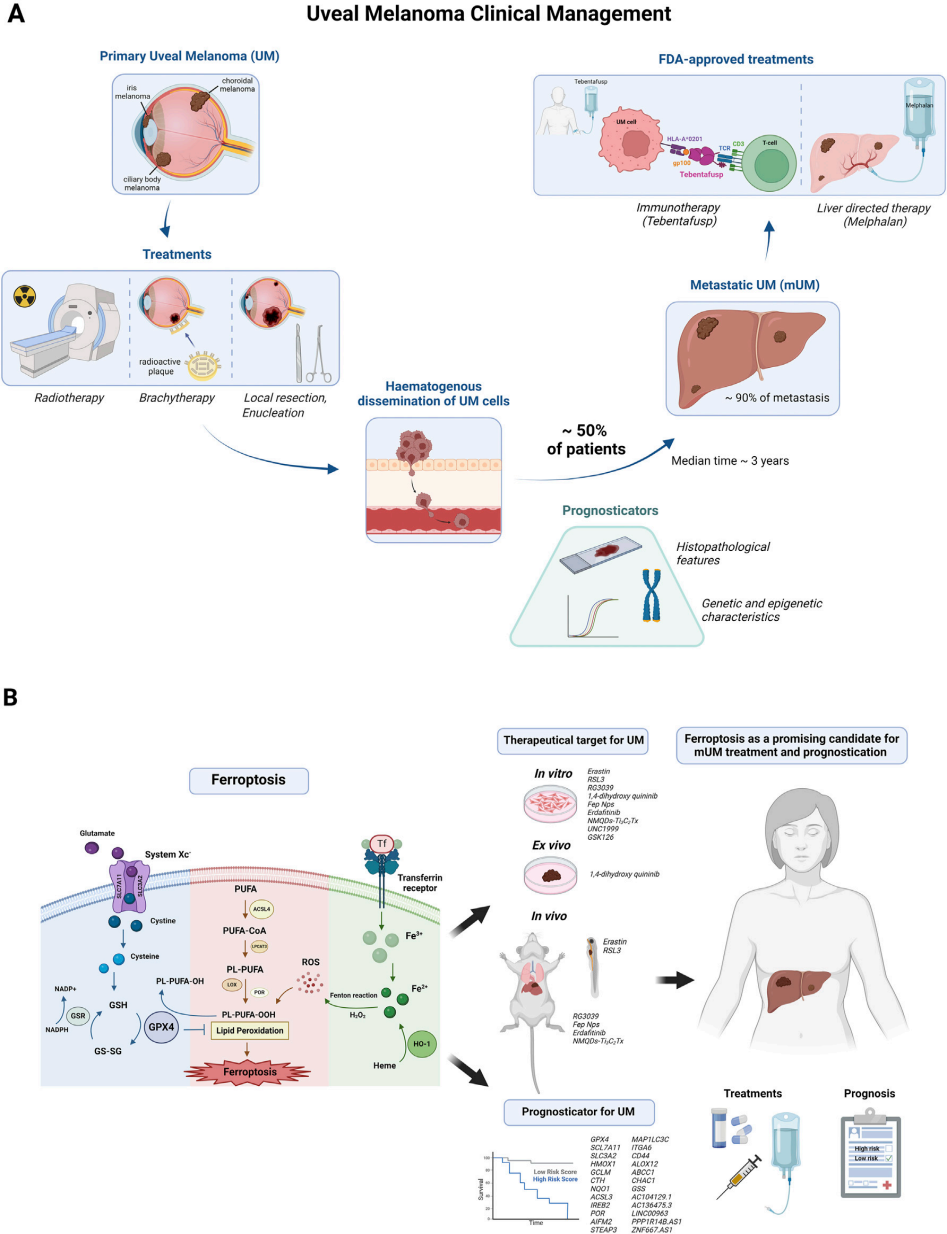
Current clinical management of uveal melanoma and evidence on ferroptosis as a potential treatment and prognosticator for the disease. **(A)** Uveal melanoma (UM) is the most common intraocular malignancy in adults, originating from melanocytes in the uveal tract (choroid, ciliary body, iris). The primary disease is typically treated with radiotherapy, which uses high-energy x-rays or a beam of high energy protons (proton beam therapy) to kill cancer cells; brachytherapy, where a plaque with radioactive sources is surgically implanted on the sclera overlying the tumor; local resection, an alternative eye-salvaging approach to the more utilized irradiation techniques; or enucleation, in which the eye is removed if the tumor is too large for vision-sparing treatments. Despite these treatments, hematogenous spread of UM cells leads to metastasis in up to half of all patients, usually within a median time of approximately 3 years. Nearly 90% of metastases occur in the liver. Histopathological features and DNA/RNA-based tests (e.g., chromosome 3 status, gene expression analysis) can predict progression to metastatic disease. However, these assays often require biopsies in patients not undergoing enucleation. Biopsies are highly invasive, carry a risk of vision-threatening complications, and are limited by sampling variability. Therapeutic options for metastatic UM are limited and include immunotherapy and liver-directed therapies. Only two FDA-approved treatments, tebentafusp-tebn and melphalan/Hepatic Delivery System (HDS), are available for metastatic UM, and these only moderately prolong survival (Jager et al., 2020; Nathan et al., 2021; Zager et al., 2022). Tebentafusp, which is administered intravenously, is a bispecific fusion protein with two key components: one binds specifically to the gp100 antigen on UM cells, while the second attaches to cluster of differentiation 3 (CD3), a receptor on T cells. This connection activates the T cells, redirecting them to attack the tumor cells. gp100: glycoprotein 100; HLA-A: human leucocyte antigen-A; TCR: T cell receptor. The melphalan/HDS is a chemotherapy drug/device combination used for liver-directed treatment of mUM patients (Zager et al., 2022). **(B)** Ferroptosis has emerged as a new player in anticancer therapies. Three main pathways regulate the process of ferroptosis: the canonical GPX4-regulated pathway, the lipid metabolism pathway and the iron metabolism pathway. Key components include system Xc^−^: cystine/glutamate antiporter; GSH: glutathione; GPX4: glutathione peroxidase 4; GS-SG: glutathione disulfide; GR: glutathione reductase; NADPH: nicotinamide adenine dinucleotide phosphate; PUFA: polyunsaturated fatty acid; ACSL4: acyl-CoA synthetase long chain family member 4; CoA: coenzyme A; LPCAT3: lysophosphatidylcholine acyltransferase 3; PL: phospholipid; LOX: lipoxygenases; POR: cytochrome P450 oxidoreductase; PL-PUFA-OOH: PUFA-containing-phospholipid hydroperoxides; PL-PUFA-OH: PUFA-containing-phospholipid alcohols; ROS: reactive oxygen species; Fe^3+^: ferric ion; Fe^2+^: ferrous ion; H_2_O_2_: hydrogen peroxide; HO-1: heme oxygenase (Zhang C. et al., 2022). Studies have demonstrated the potential of ferroptosis as a therapeutic target in UM through various treatments *in vitro* (Erastin, RSL3, RG3039, 1,4-dihydroxy quininib, Fep Nps, Erdafitinib, NMQDs-Ti3C2Tx, UNC 1999); *ex vivo* (1,4-dihydroxy quininib); and *in vivo* (RG3039, Fep Nps, Erdafitinib, NMQDs-Ti3C2Tx in mouse UM models; Erastin and RSL3 in zebrafish UM models). Ferroptosis-related signatures including *GPX4*, *SCL7A11*, *SLC3A2*, *HMOX1*, *GCLM*, *CTH*, *NQO1*, *ACSL3*, *IREB2*, *POR*, *AIFM2*, *STEAP3*, *MAP1LC3C*, *ITGA6*, *CD44*, *ALOX12*, *ABCC1*, *CHAC1*, *GSS*, *AC104129.1*, *AC136475.3*, *LINC00963*, *PPP1R14B.AS1*, and *ZNF667.AS1* appear to be good candidate to predict disease progression and response to therapies (Dörschmann et al., 2021; Groenewoud et al., 2023; Hou et al., 2022; Jin et al., 2023; Tonelotto et al., 2024; Wang et al., 2024; Zhang H. et al., 2022; Zhu et al., 2024). Created with BioRender.com. Subscription: Institution (University College Dublin).

Uveal melanoma (UM) is the most common primary intraocular tumour in adults (Jager et al., 2020). The incidence of UM varies globally: Northern Europe, Western Europe, and Oceania have the highest incidence (>8 per million per year), followed by North America, Eastern Europe, and Southern Europe (2-7.9 per million per year), with South America, Asia and Africa having the lowest incidence (<2 per million per year) (Gelmi and Jager, 2024; Singh et al., 2011; Virgili et al., 2007; Wu et al., 2023). UM arises from melanocytes in the uvea, consisting of the pigment tissues of the iris (4% of cases), the ciliary body (6% of cases), and the choroid (90% of cases) (Shields et al., 2009). Risk factors for UM include older age, fair skin, light eyes, inability to tan, ocular or oculodermal melanocytosis, cutaneous, iris or choroidal nevi and BRCA-1 associated protein 1 (*BAP1*) mutations (Jager et al., 2020). Men have a higher age-adjusted UM incidence (5.8 per million) compared to women (4.4 per million) (Singh et al., 2011; Jager et al., 2020). When present, UM symptoms include blurred or distorted vision, visual field loss, and photopsia (Damato and Damato, 2012). Clinical diagnosis of UM is typically conducted using indirect ophthalmoscopy and eye slit lamp biomicroscopy (Jager et al., 2020). Here, we discuss current strategies for the clinical management of UM (Figure 1A), and explore ferroptosis as a promising candidate for UM treatment and prognostication (Figure 1B; Table 1).

**TABLE 1:** List of the *in vitro*, *ex vivo* and *in vivo* studies conducted on ferroptosis in uveal melanoma.

Drug	Role in ferroptosis induction	*In vitro, ex vivo* or *in vivo* testing	Results	References
RG30309	DCPS inhibition induces GLRX-mediated ROS production, leading to the formation of lipid peroxides	*In vitro:* UM 92.1 (primary), Mel270 (primary), OMM1 (metastatic), OMM2.3 (metastatic) and MP41 (primary) cells *In vivo:* NOD/SCID mice injected subcutaneously with OMM1 cells	RG30309 impedes UM cell growth *in vitro* through ferroptosis and diminishes hepatic metastasis of UM cells *in vivo*	Jin et al. (2023)
1,4-dihydroxy quininib	- Increases ROS, HO-1 expression and lipid hydroxide levels - Decreases GPX4 expression and affects GSH content - Modulates the expression of ferroptosis-related proteins including SLC3A2, GCLM, CTH, ACSL3, NQO1, IREB2 and AIFM2	*In vitro:* UM OMM2.5 (metastatic) and Mel285 (primary) cells *Ex vivo:* Metastatic UM OPDX	1,4-dihydroxy quininib induces UM cell death through ferroptosis *in vitro* and increases ferroptotic markers levels *ex vivo*	Tonelotto et al. (2024)
Gallic acid (GA)-Fe (III) and paclitaxel (PTX)-assembled nanoparticles (Fep Nps)	- Increase ROS, membrane lipid peroxidation and intracellular iron levels - Suppresse intracellular GSH production by inhibiting system Xc^-^ activation - Decrease GPX4 expression and increase ACSL4 levels	*In vitro:* B16F10 cells (from mouse melanoma) *In vivo:* Nude mice injected with B16F10-Luc melanoma cells intraocularly in the right eye	FepNps reduce melanoma cells growth *in vitro* by ferroptosis induction and inhibits tumor growth and metastasis *in vivo*	Wang et al. (2024)
Erdafitinib	- Affects iron, GSH and MDA levels - Enhances the transcriptional activity of TFEB, whose aggregation in the nucleus triggers FTH1-dependent ferritinophagy, leading to lysosomal activation	*In vitro:* UM 921.1 (primary) and Mel202 (primary) cells *In vivo:* UM 92.1 xenograft models	Erdafitinib induces UM cell death by promoting ferroptosis *in vitro* and suppresses tumor growth *in vivo*	Zhu et al. (2024)
NMQDs-Ti_3_C_2_Tx	- Increases ROS, MDA and lipid peroxide levels - Decreases GSH concentration and increases *SLC7A11* levels - Upregulates *PTGS2* levels - Induces mitophagy and lysosome destruction	*In vitro:* UM C918 (primary) and Mum-2B (metastatic) cells *In vivo:* Mice injected with UM C918 cells	NMQDs-Ti_3_C_2_Tx evokes ferroptosis in UM cells *in vitro* and prevents UM xenograft tumor growth *in vivo*	Zhang et al. (2022a)
UNC 1999	- Increases intracellular iron, ROS and MDA levels; decreases GSH levels - Modulates expression of genes involved in ferroptosis pathways including *HMOX1, NRF2, TXNIP, FITH1, SQSTM1* and *KEAP1* *-* Causes structural damage and dysfunction in mitochondria	*In vitro:* UM OMM1 cells (metastatic)	UNC 1999 suppresses UM cell growth *in vitro* through ferroptosis	Hou et al. (2022)
Erastin and RSL3	- Erastin: SCL7A11 inhibitor - RSL3: GPX4 inhibitor	*In vitro:* UM MM66 (metastatic) and Mel285 (primary) cells *In vivo:* Patient-derived zebrafish xenografts of primary and metastatic UM	Erastin and RSL3 reduces UM cell growth *in vitro* and diminishes metastasis formation *in vivo*	Groenewoud et al. (2023)
Erastin	Decreases GPX4 expression	*In vitro:* UM OMM1 (metastatic) cells	Erastin induces UM cell death *in vitro,* which can be rescued by specific fucoidan extracts	Dörschmann et al. (2021)

### 1.1 Uveal melanoma clinical management

Local resection, radiation therapy (including plaque radiotherapy and proton beam therapy), and enucleation are the current treatments for primary UM (Yang et al., 2018). However, regardless of the treatment received, many patients develop metastatic UM (mUM) within 5 to 7 years (Awh and Wilson, 2020). The median survival time after diagnosis of metastasis in the largest published series of unselected patients with mUM was 3.6 months, with less than 1% of patients surviving after 5 years (Diener-West et al., 2005). This poor prognosis significantly impacts the quality of life and mental wellbeing of UM patients. UM patients with unfavourable prognoses, as determined by cytogenetic testing, show increased depression and anxiety, compared to patients with favourable prognoses (Rabsahl et al., 2023). Furthermore, UM patients with metastases have been reported to have elevated anxiety and depression compared to those without metastases (Rabsahl et al., 2023).

Common sites of metastasis include the liver, lung, bone, skin, subcutaneous tissue and the lymph nodes (Diener-West et al., 2005). In 2022, tebentafusp-tebn (KIMMTRAK^R^) was approved by the Food and Drug Administration (FDA) and the European Medicines Agency (EMA), as the first disease-specific treatment for mUM, but tebentafusp-tebn is restricted to HLA-A*0201 haplotypes, representing 50% of patients with European ancestry (Hurley et al., 2020; Chen and Carvajal, 2022; Nathan et al., 2021). In August 2023, the FDA approved HEPZATO KIT (melphalan for injection via the hepatic delivery system) for mUM. To be eligible for melphalan patients must have unresectable metastases in less than half of the liver and extrahepatic disease limited to the bone, lymph nodes, subcutaneous tissues or lung that is amenable to resection or radiation (Zager et al., 2022). This criterion may leave patients with advanced metastatic disease unable to receive melphalan. Recently, the combination of crizotinib and darovasertib showed promising results in clinical trials; however, the combination has yet to receive FDA approval (Cao et al., 2023).

Clinicians use histopathological features and genetic aberrations for UM prognosis. Poor prognostic factors include large tumour size, ciliary body involvement, extra-scleral extension, the presence of epithelioid cells compared to spindle cells, a high number of mitotic figures in the tumour, presence of extravascular loops and networks, and vortex vein involvement (Kaliki et al., 2015). The driver UM mutations occur in G protein subunit alpha q (*GNAQ*), G protein subunit alpha 11 (*GNA11*), cysteinyl leukotriene receptor 2 (*CYSLTR2*), or phospholipase C beta 4 (*PLCB4*), which activate the G alpha q pathway (Gelmi and Jager, 2024), but these mutations are typically not prognostic. A chromosomal aberration associated with poor prognosis is chromosome 3 loss, leading to *BAP1* inactivation, which occurs in 84% of metastasizing UMs (Harbour et al., 2010). UM patients with disomy 3 have a 90% 5-year survival rate, compared to 37% for patients with monosomy 3 (Versluis et al., 2015). Gene expression profiling (GEP) and Preferentially Expressed Antigen in Melanoma (PRAME) expression are also used for prognostication, with GEP class 2 tumours and high PRAME levels indicating worse outcomes (Gelmi and Jager, 2024). The Liverpool Uveal Melanoma Prognosticator Online algorithm can determine the risk of metastases and estimate UM patient survival (Lamas et al., 2021).

Epigenetic mechanisms, including DNA methylation, chromatin remodelling, histone modification, and non-coding RNAs (miRNAs), also play a role in predicting prognosis for UM patients (Chokhachi Baradaran et al., 2020). One example is the Ras association domain family 1 isoform A (*RASSF1A*) gene, which is often silenced through promoter hypermethylation in UM, promoting cancer progression (Calipel et al., 2011). Methylation of the *P16INK4A* promoter is also associated with a higher risk of metastasis in UM patients (Maat et al., 2008). Additionally, increased expression of certain miRNAs (let-7b, miR-143, miR-193b, miR-199a, and miR-652) is linked to more aggressive Class 2 UM tumours, helping distinguish them from less aggressive Class 1 tumours (Worley et al., 2008).

Ongoing UM research aims to develop gene signatures to create reliable prognostic models, as no universally accepted model combining clinical and molecular data currently exists.

### 1.2 Ferroptosis has potential as a novel therapeutic target for UM

Ferroptosis is a form of cell death triggered by iron-mediated overproduction of lipid reactive oxygen species (ROS). It is morphologically and biochemically distinct from autophagy, apoptosis, necrosis, and necroptosis (Dixon et al., 2012). Ferroptosis can be induced by inhibiting cell membrane transporters such as the cystine/glutamate transporter (system Xc^−^), by activating the iron transporters serotransferrin and lactotransferrin, or by blocking intracellular antioxidant enzymes, such as glutathione peroxidase 4 (GPX4) (Tang and Kroemer, 2020). Normally, the cystine/glutamate transporter brings cystine into cells, where it is converted to cysteine, which is used to produce glutathione (GSH). GPX4 uses GSH to convert polyunsaturated-fatty-acid-containing-phospholipid hydroperoxide (PL-PUFA-OOH) into phospholipid alcohols, preventing PL-PUFA-OOH accumulation which induces ferroptosis (Yang et al., 2014; Zhang C. et al., 2022). Cellular iron uptake occurs through serum transferrin binding to the transferrin receptor and subsequent endocytosis. A build-up of iron intracellularly generates ROS through the iron-dependent Fenton reaction, initiating lipid peroxidation and thus ferroptosis (Dixon and Stockwell, 2014; Zhang H. et al., 2022). Iron accumulation, and consequently ferroptosis induction, can also result from the enzymatic activity of heme oxygenase 1 (HO-1), which breaks down heme into carbon monoxide, biliverdin, and free iron (Chiang et al., 2018).

Ferroptosis reduces metastasis in several cancers and can overcome treatment resistance (Chen et al., 2023; Wang et al., 2023; Zhang C. et al., 2022). Examples include cisplatin, which induces ferroptosis by inhibiting system Xc^-^ and GPX4, and enhances the effects of immune checkpoint therapy in non-small cell lung cancer (Zhou et al., 2022). In triple-negative breast cancer, cells resistant to gefitinib become more sensitive when GPX4 is inhibited, which leads to ferroptosis (Song et al., 2020). In KRAS-mutant metastatic colorectal cancer, combining the ferroptosis inducer β-elemene with cetuximab increases sensitivity to treatment by triggering ferroptosis (Chen et al., 2020). Research shows that ferroptosis contributes to the death of 90%–99% of circulating cancer cells, preventing them from metastasizing (Groenewoud et al., 2023; Piskounova et al., 2015; Yagoda et al., 2007). Therefore, inducing ferroptosis in resistant tumours with a high propensity to metastasize, such as UM, may be beneficial. Intriguingly, ferroptosis is the cell death mechanism of several anti-cancer drugs, highlighting its potential (Su et al., 2020). Furthermore, analyses of the Tissue Cancer Genome Atlas (TCGA) and Gene Expression Omnibus (GEO) datasets show that ferroptosis-associated genes can predict UM prognosis (Groenewoud et al., 2023; Jin et al., 2021; Luo and Ma, 2021; Ma et al., 2022; Tonelotto et al., 2024; Wang et al., 2022).

## 2 Evidence on ferroptosis as a promising strategy for uveal melanoma treatment and prognostication

Recent studies have shown the effectiveness of ferroptosis in *in vitro*, *ex vivo* and *in vivo* UM models, which are discussed below (Table 1; Figure 1B).

### 2.1 *In Vitro* efficacy of ferroptosis in uveal melanoma

Jin et al. proposed that decapping scavenger enzymes (DCPS) impact ferroptosis by regulating mRNA decay during UM metastasis to the liver (Fuchs et al., 2020; van Dijk et al., 2003). This hypothesis is based on the fact that DCPS regulates expression of glutaredoxins (GLRX), which scavenge ROS, thereby preventing ferroptosis. The group treated UM cells with RG30309, a DCPS inhibitor, which resulted in an increase in ROS generation and ferroptosis induction. (Jin et al., 2023).

Tonelotto et al. (2024) demonstrated the *in vitro* efficacy of ferroptosis in UM by elucidating the mechanism of action of 1,4-dihydroxy quininib, a cysteinyl leukotriene receptor 1 (CysLT_1_) antagonist which inhibits UM hallmarks (Slater et al., 2022; Slater et al., 2020; Tonelotto et al., 2024). The group performed proteome-profiling of OMM2.5 mUM cell extracts treated with 1,4-dihydroxy quininib. They found that HO-1 was consistently upregulated, with lipid hydroperoxide and ROS levels increasing and GPX4 expression decreasing in a time-dependent manner. These findings indicate that 1,4-dihydroxy quininib reduces UM cell viability by inducing ferroptosis (Tonelotto et al., 2024). Treatment of OMM2.5 cells with the histone deacetylase inhibitor (HDACi) ACY-1215 or with the natural product Ergolide, both of which inhibit UM hallmarks, also altered the expression of ferroptotic markers ASCL3 and GCLC or HO-1 and GDF15, respectively (Sundaramurthi et al., 2023; Sundaramurthi et al., 2022). This reinforces ferroptosis as a UM therapeutic target.

Wang and colleagues developed gallic acid (GA)-Fe (III) and paclitaxel (PTX)-assembled nanoparticles (FeP Nps), which are internalized into tumour cells through ultrasound (US) irradiation, activating the Fenton reaction and leading to ferroptosis (Wang et al., 2024). FepNPs also inhibited solute carrier family member 7 (SLC7A11) thus suppressing GSH production; decreased GPX4 levels; and increased the expression of Acyl-CoA synthase long-chain family member 4 (ACSL4), which modulates lipid composition levels (Ding et al., 2023; Wang et al., 2024). Furthermore, the treated cells displayed altered mitochondria (Wang et al., 2024). Overall, these results indicate that FeP NPs activate ferroptosis. It is important to note that B16F10 cells are melanoma cells, which raises questions about this treatment’s efficacy in UM.

Zhu and co-authors targeted the fibroblast growth factor (FGF)/FGF Receptor (FGFR) axis, which is associated with poor prognosis in UM patients (Rezzola et al., 2019). Erdafitinib, a pan-FGFR tyrosine kinase inhibitor, reduced 92.1 and Mel202 UM cell viability (Zhu et al., 2024). Additionally, Erdafitinib affected the content of iron and GSH, as well as the levels of malondialdehyde (MDA), thus implicating ferroptosis (Cordiano et al., 2023; Zhu et al., 2024).

Zhang H. et al. (2022) evaluated the effect of non-oxidized MXene-Ti_3_C_2_Tx quantum dots (NMQDs-Ti_3_C_2_Tx) on C918 (UM) and Mum-2B (mUM) cell lines (Zhang C. et al., 2022). Following the treatment, cell proliferation and invasiveness were reduced, and intracellular levels of ROS, MDA, and lipid peroxides increased. The treatment also decreased intracellular GSH concentration and upregulated RNA levels of *SLC7A11*, indicating negative feedback on GSH. Moreover, NMQDs-Ti_3_C_2_T_x_ treatment upregulated mRNA levels of *PTGS2*, a ferroptosis-related gene (Zhang H. et al., 2022). These findings indicate that NMQDs-Ti_3_C_2_Tx evoke UM cell death through ferroptosis.

Hou's group investigated the effect of small-molecule inhibitors of enhancers of zeste homolog 2, including UNC 1999 and GSK126, in OMM1 mUM cells. They found that UNC 1999 and GSK126 suppressed OMM1 mUM cell growth, with UNC 1999 altering the expression of ferroptosis-related genes. In addition, UNC 1999 increased intracellular Fe^2+^, ROS and MDA levels, decreased GSH levels, disrupted mitochondria morphology, and diminished maximal respiration and adenosine triphosphate production in the mitochondria. These findings provide compelling evidence that UNC 1999 induces UM cell death through ferroptosis (Hou et al., 2022).

Research conducted by Groenewoud et al. showed that the ferroptosis inducers erastin and RSL3 reduced MM66 (mUM) and Mel285 (UM) cell viability (Groenewoud et al., 2023); while Dörschmann et al. demonstrated that erastin induced OMM1 cell death, and that specific fucoidan extracts inhibited erastin effects (Dörschmann et al., 2021).

### 2.2 *Ex Vivo* and *in Vivo* efficacy of ferroptosis in uveal melanoma

Ferroptosis has been thoroughly investigated in *ex vivo* and *in vivo* UM models, which provide greater insight into the tumour macro- and microenvironment than cell lines (Hidalgo et al., 2014; Lee et al., 2021).

Jin et al. subcutaneously injected OMM1 cells into Non-Obese Diabetic Severe Combined Immune Deficiency (NOD/SCID) mice and, after 3 weeks, observed that the tumour volumes and weights of OMM1 xenografts were significantly reduced after RG3039 treatment compared to the vehicle-treated mice (Jin et al., 2023). Additionally, levels of 4-hydroxynonenal (4-HNE), a reactive aldehyde derived from lipid peroxidation, increased after RG3039 treatment. In a separate set of mice bearing UM patient-derived xenografts (PDX), RG3039 treatment similarly reduced tumour volume and weight (Jin et al., 2023). This research supports the *in vivo* efficacy of ferroptosis in UM.

Tonelotto and colleagues treated tumour explants from mUM orthotopic PDX mouse models with 1,4-dihydroxy quininib. The treatment led to an upregulation of HO-1 expression and to increased levels of 4-HNE, suggesting that 1,4-dihydroxy quininib induces ferroptosis in patient-derived mUM explants (Tonelotto et al., 2024).

Wang et al. observed a near-complete disappearance of intraocular tumours in nude mice injected with BF16F10-Luc melanoma cells intraocularly and treated with Fep Nps and US irradiation (Wang et al., 2024). These results provide evidence for the anticancer efficacy of Fep Nps, which induced ferroptosis *in vitro*.

Zhu's group treated 92.1 xenograft models with Erdafitinib, which reduced lesion volume compared to the control group. These results align with *in vitro* data demonstrating Erdafitinib’s anticancer activity through ferroptosis induction (Zhu et al., 2024).

Zhang et al. (2022b) found that tumor growth significantly increased in the control group compared to the NMQDs-Ti_3_C_2_Tx-treated group in mice injected with C918 cells (Zhang et al., 2022b). This supports the antitumor activity of NMQDs-Ti_3_C_2_Tx, which were shown to induce ferroptosis *in vitro*.

Groenewoud's team generated patient-derived zebrafish UM xenograft models and treated them with erastin and RSL3. RSL3 inhibited metastasis formation in zebrafish engrafted with UM and mUM cells, all of which highly expressed GPX4. Similarly, erastin inhibited tumour burden in zebrafish engrafted with UM and mUM cells (Groenewoud et al., 2023), highlighting the *in vivo* efficacy of ferroptosis in UM.

### 2.3 Ferroptosis gene signatures as a uveal melanoma prognostic tool

Ferroptosis-related gene signatures have emerged as potential biomarkers for UM, offering novel insights into prognosis and treatment response.

Groenewoud et al. (2023) found that *GPX4* expression negatively correlates with survival in the Leiden University Medical Centre (LUMC) UM cohort, while *SLC7A11* expression shows similar trends in the TCGA-UM cohort. They also analyzed a panel of eight primary UM patient samples, which were compared to a *BAP1* positive and *BAP1* negative mUM cell lines. The analysis revealed a strong inverse correlation between *BAP1* and *GPX4*, implicating ferroptosis in mUM progression.

Tonelotto et al. (2024) analysed data from the TCGA-UM and GSE84976 cohorts and identified a ferroptosis-related gene signature (IFerr), including *GPX4*, *SCL7A11*, solute-carrier family 3 member 2 (*SLC3A2)*, glutamate cysteine ligase modifier (*GCLM)*, cystathionine gamma-lyase (*CTH*), acyl-CoA synthetase long-chain family member 3 (*ACSL3*)*,* NAD(P)H quinone dehydrogenase 1 (*NQO1*), iron responsive element binding protein 2 (*IREB2*) and apoptosis inducing factor mitochondria associated 2 (*AIFM2*). High IFerr is observed in monosomy 3 patients in both databases and predicts decreased overall survival (OS) and disease-free survival (DFS) in UM patients.

Luo and Ma (2021) developed a ferroptosis-related gene signature including six-transmembrane epithelial antigen of prostate 3 (*STEAP3)*, microtubule associated protein 1 light chain 3 gamma (*MAP1LC3C)*, integrin subunit alpha 6 (*ITGA6)*, *HO-1*, *CD44*, arachidonate 12-lipoxygenase (*ALOX12)* and AIFM2/ferroptosis suppressor protein1 (*FSP1)* using data from TCGA-UM and GSE22138 cohorts. This signature is strongly associated with UM prognosis and can precisely detect UM risk level.

The research from Jin et al. (2021) identified that the expression of ferroptosis regulators including *ABCC1*, glutathione specific gamma-glutamylcyclotransferase 1 (*CHAC1)* and glutathione synthetase (*GSS)*, is associated with poor OS, PFS and disease-specific survival (DSS) in UM.

Ma et al. (2022) evaluated ferroptosis-related long noncoding RNAs (FRLs) using the TGCA-UM database and identified a five FRLs signature, including *AC10412*9.1, *AC136475.3*, *LINC00963*, *PPP1R14B.AS1* and *ZNF667.AS1*, which correlates with a shorter overall life expectancy in UM patients.

Wang et al. (2022) found significant correlations between the expression levels of transient receptor potential melastatin 4 (TRPM4) and transient receptor potential vanilloid 2 (TRPV2) with OSS and DSS in the TCGA-UM cohort. They also observed that expression levels of farnesyl-diphosphate farnesyltransferase 1 *(FDFT1),* solute carrier family 1 member 5 *(SLCIA5),* heat shock proteins *(HSPAS), SLC7A11* and ER membrane protein complex subunit 2 *(EMC2)*, which participate in ferroptosis, significantly correlate with TRPV2 and TRPM4 expression. This suggests that TRPM4 and TRPV2 are associated with the ferroptosis pathway in UM.

## 3 Current challenges of implementing ferroptosis inducers in the clinic

### 3.1 Immune implications of ferroptosis inducers

Inducing ferroptosis as a cancer treatment presents several challenges and potential side effects that must be carefully considered. One major concern is its impact on immune cells, where ferroptosis in CD8^+^ T cells can weaken antitumor immunity, as observed in colon cancer models. This effect, though mitigated by overexpressing GPX4, underscores the potential for ferroptosis to negatively affect the immune response (Ma et al., 2021; Xu et al., 2021). Furthermore, the ferroptosis inducer RSL3 impairs dendritic cell function, suggesting risks to immune system integrity (Han et al., 2021).

### 3.2 Potential side effects of ferroptosis inducers

Cardiovascular and neurological risks are also associated with ferroptosis induction. For example, reduced GPX4 and increased iron levels have been linked to heart disease progression and are implicated in neurological conditions like hemorrhagic stroke (Baba et al., 2018; Nobuta et al., 2019; Zille et al., 2017).

Gastrointestinal and renal complications further complicate ferroptosis treatment. For instance, ferroptosis can exacerbate inflammatory bowel disease and trigger acute kidney injury when GPX4 is depleted (Mayr et al., 2020; Friedmann Angeli et al., 2014).

Cancer-specific challenges also exist. In pancreatic cancer, ferroptosis can promote tumor growth by influencing macrophage polarization and is linked to conditions like pancreatitis and pancreatic tumorigenesis (Dai et al., 2020; Liu et al., 2022). It may also worsen hepatocellular carcinoma development (He et al., 2023).

Additionally, resistance to ferroptosis inducers is a potential issue, as seen in mUM cells that increase GLCM expression and glutathione content to resist ferroptosis (Tonelotto et al., 2024).

While ferroptosis holds promise as a cancer treatment, addressing these challenges, including specificity, toxicity, resistance, and immune effects, is essential for its successful clinical application. These obstacles could potentially be overcome by ensuring that ferroptosis inducers selectively target cancer cells, minimizing damage to healthy tissues. One promising approach is the use of nanotechnology to deliver ferroptosis inducers directly to tumours, enhancing specificity and reducing systemic toxicity (Xiang et al., 2024). Additionally, combination therapies that pair ferroptosis inducers with existing treatments, like immunotherapy or chemotherapy, may help increase treatment efficacy while managing side effects (Huang et al., 2023). Further research into biomarkers for ferroptosis sensitivity could also allow for more personalized treatment strategies.

## 4 Discussion

Ferroptosis inducers represent a promising new approach for treating mUM, addressing the limitations of current therapies. These inducers trigger a unique form of cell death that operates independently of other treatment mechanisms, such as those targeted by darovasertib, a PKC inhibitor, and crizotinib, a c-Met inhibitor, which have shown significant efficacy in reducing tumor size in Phase II trials (Cao et al., 2023). Ferroptosis inducers could thus be valuable when resistance to these targeted therapies occurs. Additionally, emerging treatments like HDAC inhibitors, especially those targeting HDAC6, are showing promise for mUM (Li, Tian, and Zhu, 2020; Sundaramurthi et al., 2022). Combining these inhibitors with ferroptosis inducers may provide a synergistic therapeutic approach. Furthermore, ferroptosis inducers may offer treatment options for patients who are ineligible for current FDA-approved therapies. Expanding ferroptosis inducers from preclinical success to clinical use requires extensive research to assess efficacy and safety through human trials, typically a decade-long process (Lee et al., 2018; Rang and Hill, 2013; Sedgwick, 2011). Comparing ferroptosis inducers with existing treatments is crucial to establish their therapeutic advantage.

In summary, ferroptosis inducers offer a novel and potentially transformative treatment for mUM, complementing and possibly enhancing the current therapeutic landscape.

## 5 Future perspectives

Overall, this review highlights the need for more translational research on ferroptosis in clinically relevant UM models to bridge the gap between preclinical findings and clinical applications.

To translate ferroptosis inducers into clinical practice, key challenges include improving delivery to tumor sites due to their limited bioavailability (Ko et al., 2024). Advances in nanotechnology offer promising solutions, such as biomimetic self-assembling nano-prodrugs that deliver multiple agents simultaneously. Huang et al. demonstrated a nano-prodrug system delivering gefitinib, ferrocene, and dihydroartemisinin effectively inhibiting tumor growth through combined ferroptosis and apoptosis therapy (Huang et al., 2023). This underscores the potential of such delivery systems and drug combinations in optimizing ferroptosis-based treatments for UM.

Ongoing translational research using clinically relevant UM models, along with addressing the challenges outlined, will facilitate the successful integration of ferroptosis into clinical cancer care, paving the way for the creation of personalized, ferroptosis-based anticancer therapies.
